# Effects of *FSHR* and *FSHB* Variants on Hormonal Profile and Reproductive Outcomes of Infertile Women With Endometriosis

**DOI:** 10.3389/fendo.2021.760616

**Published:** 2021-09-30

**Authors:** Bianca Bianco, Flavia Altheman Loureiro, Camila Martins Trevisan, Carla Peluso, Denise Maria Christofolini, Erik Montagna, Antonio Simone Laganà, Caio Parente Barbosa

**Affiliations:** ^1^ Discipline of Sexual and Reproductive Health, and Populational Genetics – Department of Collective Health, Faculdade de Medicina do ABC/Centro Universitário FMABC, Santo André, Brazil; ^2^ Department of Urology, Instituto Israelita de Ensino e Pesquisa Albert Einstein, São Paulo, Brazil; ^3^ Postgraduate Program in Health Sciences, Faculdade de Medicina do ABC/Centro Universitário FMABC, Santo André, Brazil; ^4^ Department of Obstetrics and Gynecology, “Filippo Del Ponte” Hospital, University of Insubria, Varese, Italy

**Keywords:** endometriosis, FSHB, FSHR, *in vitro* fertilization, single nucleotide variant

## Abstract

**Background:**

Single nucleotide variants (SNVs) *FSHB*:c.-211G>T, *FSHR*:c.919G>A, and *FSHR*:c.2039G>A were reported to be associated with the variability in FSH and LH levels, and *in vitro* fertilization (IVF) outcomes. In this study, we aimed to evaluate the effects of *FSHB*:c.-211G>T, *FSHR*:c.919G>A, and *FSHR:*c.2039G>A variants, alone and combined, on the hormonal profile and reproduction outcomes of women with endometriosis.

**Methods:**

A cross-sectional study was performed comprising 213 infertile Brazilian women with endometriosis who underwent IVF treatment. Genotyping was performed using TaqMan real-time PCR. Variables were compared according to the genotypes of each variant and genetic models, and the combined effects of the SNVs were evaluated using the multifactorial dimensionality reduction method.

**Results:**

*FSHB*:c.-211G>T affected LH levels in women with overall endometriosis and minimal/mild disease. *FSHR*:c.919G>A affected FSH levels in women with overall endometriosis and the number of oocytes retrieved in those with moderate/severe endometriosis. Moreover, the *FSHR*:c.2039G>A affected FSH levels in women with overall endometriosis, LH levels and total amount of rFSH in those with minimal/mild disease, and number of follicles and number of oocytes retrieved in those with moderate/severe endometriosis. No effect on hormone profile or reproductive outcomes was observed when the genotypes were combined.

**Conclusions:**

Variants of the *FSHB* and *FSHR* genes separately interfered with the hormonal profiles and IVF outcomes of women with endometriosis.

## Background

Endometriosis is one of the most common diseases associated with infertility, affecting 5–10% women of reproductive age. Endometriosis is heterogeneous in presentation, and its pathogenesis remains still elusive (1). Among the different subtypes, deep infiltrating endometriosis and ovarian endometriomas are associated with a clear fertility impairment (2), whereas less robust data are available for peritoneal superficial lesions (3).

Follicle-stimulating hormone (FSH) is essential for the hypothalamic-pituitary-gonadal axis and plays a key role in human reproductive processes, such as follicle development, oocyte maturation, regulation of steroid synthesis, granulosa cell growth, and induction of the synthesis of the androgen-converting enzyme aromatase (4). The hormone is a heterodimer comprising a hormone-specific β-chain that is associated with an α-chain and exerts its biological activities by binding to the FSH receptor (FSHR). The β-subunit encoded by the *FSHB* gene is responsible for ensuring the binding specificity to FSHR, a transmembrane glycoprotein encoded by the *FSHR* gene (5–7).

Accumulating evidence suggests that a number of single nucleotide variants (SNVs) in genes involved in FSH signaling, estrogen biosynthesis, folliculogenesis, and folate metabolism, in association with other factors, can influence the ovarian response to exogenous gonadotropins in assisted reproductive treatment (ART) (8, 9). Therefore, the molecular biology of the triggered cellular pathways for these medications has attracted more attention in clinical investigations (10). Among the variants in the coding region of *FSHR*, two (c.919G>A, p.Ala307Thr and c.2039G>A, p.Ser680Asn) have been extensively studied in ART protocols to evaluate the stimulation of FSH receptors by gonadotropins (11). These *FSHR* variants were previously associated with variability in the serum FSH levels and reproductive outcomes. They seem to influence the ovarian sensitivity to exogenous gonadotropin and to play a significant role in determining the ovarian response to controlled ovarian stimulation. So, these *FSHR* variants may influence the response to ovarian stimulation in terms of FSH consumption and duration of stimulation, and number of metaphase II oocytes retrieved after IVF treatment (12–16). In addition, an SNV in the *FSHB* gene promoter, c.-211G>T, has already been associated with lower FSH levels, age at menopause, polycystic ovary syndrome, luteinizing hormone (LH) levels, and *in vitro* fertilization (IVF) outcomes in women (13, 17–22).

Inspired by these findings, we aimed to evaluate the effects of *FSHB*:c.-211G>T, *FSHR*:c.919G>A, and *FSHR:*c.2039G>A variants, alone and combined, on the hormonal profile and reproduction outcomes of women with endometriosis.

## Methods

This cross-sectional study included 213 infertile women with endometriosis who underwent IVF treatment at the Human Reproduction and Genetics Center of the Centro Universitário Saúde ABC, Santo Andre, Brazil, between September 2014 and September 2019. The design, analysis, interpretation of data, drafting, and revisions followed the Helsinki Declaration and the strengthening the reporting of observational studies in epidemiology (STROBE) statement, available through the enhancement of the quality and transparency of health research (EQUATOR) network (www.equator-network.org). The study design was approved by the independent Research Ethics Committee of the “Centro Universitário Saúde ABC” (approval code CAEE CAAE 62507216.8.0000.0082). Each patient enrolled in this study signed an informed consent form for all procedures and to allow data and biological sample collection and analysis for research purposes. No remuneration was offered to enter or continue the study.

### Patients

The inclusion criteria were endometriosis diagnosed using laparoscopy and histological confirmation, classified according to the revised American Society for Reproductive Medicine (rASRM) score (23); age ≤38 years, FSH (≤12.0 IU/L), TSH (≥0.5 ≤ 4 IU/L), and serum prolactin levels (<25 ng/mL), body mass index (BMI; >18.5<30), ovulatory cycles (25–35 days), and the presence of both ovaries without any malformations. Patients with a history of ovarian surgery, who underwent chemo/radiotherapy, with endometrial polyps, hydrosalpinx, and submucosal and/or intramural fibroids, as well as couples whose partners underwent invasive procedures for sperm retrieval were excluded from the study.

The investigation into the cause(s) of infertility included a hormonal and biochemical profile, testing for sexually transmitted diseases, imaging examinations, investigation of genetic and/or immunological abnormalities, hysterosalpingography, hysteroscopy, laparoscopy, and semen analysis of the partner (24, 25).

We considered women who had undergone laparoscopy/laparotomy to treat endometriosis and who had failed to achieve pregnancy spontaneously or by IVF treatment after surgery within a maximum period of 12 months, as infertile.

Clinical data, hormonal profiles, and reproductive outcomes were collected from the medical records of the participants.

### Hormone Measurement

FSH and LH levels were measured during the follicular phase of the menstrual cycle, while progesterone and prolactin levels were measured during the luteal phase of the menstrual cycle.

### Antral Follicle Counting

The ovaries were evaluated before the initiation of COS on the second day of the menstrual cycle using a conventional two-dimensional transvaginal ultrasound at 7 MHz (Philips^®^). The antral follicle counting (AFC) was performed on each ovary for follicles that were ≥10 mm in length (26).

### Controlled Ovarian Stimulation

COS was performed using exogenous recombinant FSH (rFSH) at a fixed dose of 150/200 UI per day that was administered, on average, for 8 to 14 days, starting on the second or third day of the menstrual cycle. When the largest follicle reached 14 mm, the GnRH antagonist (Orgalutran^®^) was also administered until the largest follicles reached between 17 and 20 mm, as determined by transvaginal ultrasonography. At this time, the patient was administered chorionic gonadotropin (HCG-Choriomon^®^) at a dose of 5000 IU or recombinant HCG (Ovidrel^®^ 250 mcg). After 34–36 h, transvaginal ultrasound-guided follicular puncture for oocyte retrieval was performed.

A maximum of two embryos were transferred, guided by transabdominal ultrasound on the third- or fifth-day post-fertilization. Embryos were not evaluated using preimplantation genetic screening. Luteal phase support was carried out with vaginal progesterone at a dose of 600 mg/day starting on the day of oocyte retrieval. Pregnancy was confirmed by the serum beta-hCG level (>25 mIU/mL) on the 12th day after embryo transfer.

### Genotyping

Peripheral blood samples were collected in an EDTA-containing tube, and DNA was extracted from peripheral lymphocytes using the standard salting out method. Genotyping of NG_008144.1:g.4790G>T (*FSHB:*c.-211G>T, rs10835638:G>T) of the *FSHB* gene, and NM_000145.3:c.919G>A (*FSHR*:c.919G>A, rs6165:C>T, p.Ala307Thr) and NM_000145.3:c.2039G>A (*FSHR*:c.2039G>A, rs6166:C>T, p.Ser680Asn) of the *FSHR* gene was performed using the TaqMan system and real-time PCR. Assays were performed using primers, probes, and Master Mix (Thermo Fisher Scientific, Waltham, MA, USA) with 25 ng of DNA per reaction. The PCR conditions were performed as follows: 50 cycles of denaturation at 95°C for 15 s and annealing/extension at 60°C for 90 s.

### Statistical Analyses

Statistical analyses were performed using the R programming language (27). The normality of the data was assessed using the Shapiro-Wilk test. The descriptive analysis was presented by the absolute and relative frequency for the qualitative data, and by the median and 95% confidence interval (95% CI) for the quantitative data. The Hardy-Weinberg equilibrium (HWE) for each variant was verified using the chi-square test. The Mann-Whitney test was used in the dominant and recessive models, and the Kruskal-Wallis test was used in the additive model followed by the Dunn test to analyze the difference between the three genotypes on hormone levels and reproductive outcomes. The chi-square test was used to assess the association between the variants and pregnancy rate.

The combined genotypes of the studied variants and the clinical, hormonal, and reproductive variables were determined using the multifactorial dimensionality reduction (MB-MDR) method (28). MB-MDR allows the analysis of gene-gene interactions with adjustment for covariates and validation by the permutation test.

Missing values police was through median imputation. Statistical significance was set at 5%, or p <0.05.

## Results

### Patients’ Characteristics

Two hundred and thirteen women took part of this study. In this group, minimal/mild (stage I and II) endometriosis was found in 82 cases (38.5%), and moderate/severe (stage III and IV) endometriosis in 131 cases (61.5%). The clinical parameters, hormonal profile, and reproductive outcomes of the infertile women with endometriosis are shown in **Table 1**.

**Table 1:** The clinical parameters, hormonal profile and reproductive outcomes of the infertile women with endometriosis.

Variables*	Endometriosis	Minimal/Mild	Moderate/Severe
N (%)	213 (100)	82 (38.5)	131 (61.5)
Age (years old)	33 (32-34)	32 (31-34)	34 (33-34)
Menarche (years old)	12.5 (12-13)	13 (12-13)	12 (12-13)
Menstrual cycle length (days)	4 (4-5)	4 (4-4.5)	4.5 (4-5)
Menstrual cycle interval (days)	28 (28-28)	28 (28-28.5)	28 (28-28)
Infertility duration (years)	4 (3-4)	4 (3-5)	3 (3-4)
BMI (Kg/m²)	23.1 (22.8-23.8)	23 (22.5-23.8)	23.1 (22.6-24.2)
LH (mUI/mL)	4.7 (4.4-5.2)	5 (4.6-5.7)	4.5 (4.1-5.6)
Progesterone (ng/mL)	6.3 (4.0-8.7)	9.4 (2.6-12.7)	5.8 (2-7.3)
Prolactin (ng/mL)	14.5 (13-16)	13.3 (10.9-16)	15.1 (13.3-17)
FSH (UI/L)	6.9 (6.6-7.3)	6.8 (6.3-7.4)	7 (6.5-7.7)
AFC	7 (6-8)	8 (7-9)	6 (5-8)
Days of COS	10 (10-11)	10 (9-11)	10.5 (10-11)
Total dose of rFSHr (UI)	1800 (1800-2000)	1400 (1200-1800)	2000 (1800-2000)
Follicles	6 (6-7)	7 (6-9)	6 (5-7)
Oocytes retrieved	6 (5-6)	6 (6-8)	5 (4-6)
MII	5 (4-5)	5 (4-7)	5 (4-5)
Embryos	3 (2-3)	3 (2-4)	3 (2-3)
Pregnancy rate/cycle (%)	65/177 (36.7%)	28/65 (43.1%)	37/112 (33%)

*Qualitative variables were presented by absolute and relative frequency, and quantitative variables by median and 95% confidence interval. BMI, Body Mass Index; AFC, Antral Follicle Count; COS, Controlled Ovarian Stimulation; FSHr, Recombinant FSH; MII, Metaphase II oocytes.

Regarding reproductive outcomes, 35 (16.4%) women did not transfer the embryos. Among these, 13 presented a poor response to controlled ovarian stimulation (COS) (only up to 3 follicles smaller than 14mm had developed when after 6 days of ovarian stimulation with gonadotropins), with eight cycles canceled and five that did not result in embryos. Among the 16 women who showed a satisfactory response to COS, in four cases, metaphase II oocytes were not obtained for fertilization; in 10 cases, there was no development of the embryos, and two women did not transfer for personal reasons. Six women presented with excessive response (not ovarian hyperstimulation syndrome) and had not yet transferred embryos that remain frozen. Thus, the pregnancy rate was 30.5% (65/213) and considering the total of 178 participants who transferred embryos, the pregnancy rate was 36.5% (65/178).

### Single Nucleotide Variant Frequencies and Analysis

The frequencies of the genotypes of the variants studied are listed in **Table 2**. Considering the *FSHB*:c.-211G>T variant, only two women presented the homozygous variant genotype; therefore, the data was presented according to the dominant model (GG versus GT + TT). In addition, 202 out of 213 women were genotyped for this variant due to the limited sample size.

**Table 2:** The genotype frequencies of the *FSHB* and *FSHR* variants in Brazilian women with endometriosis.

	*FSHB*	*FSHR*	
	rs10835638:G>T	rs6165:C>T	rs6166:C>T
N	202	213	213
Genotypes			
WW	164 (81.2%)	56 (26.3%)	73 (34.3%)
WV	36 (17.8%)	96 (45.1%)	98 (46.0%)
VV	2 (1%)	61 (28.6%)	42 (19.7%)
Minor Allele	T	C	T
MAF	0.099	0.488	0.427
HWE	1	0.358	0.682

Genotypes: WW, Wild-type; WV, Heterozygote; VV, Variant; MAF, Minor allele frequency; HWE, Hardy-Weinberg equilibrium.

**Figure 1:**
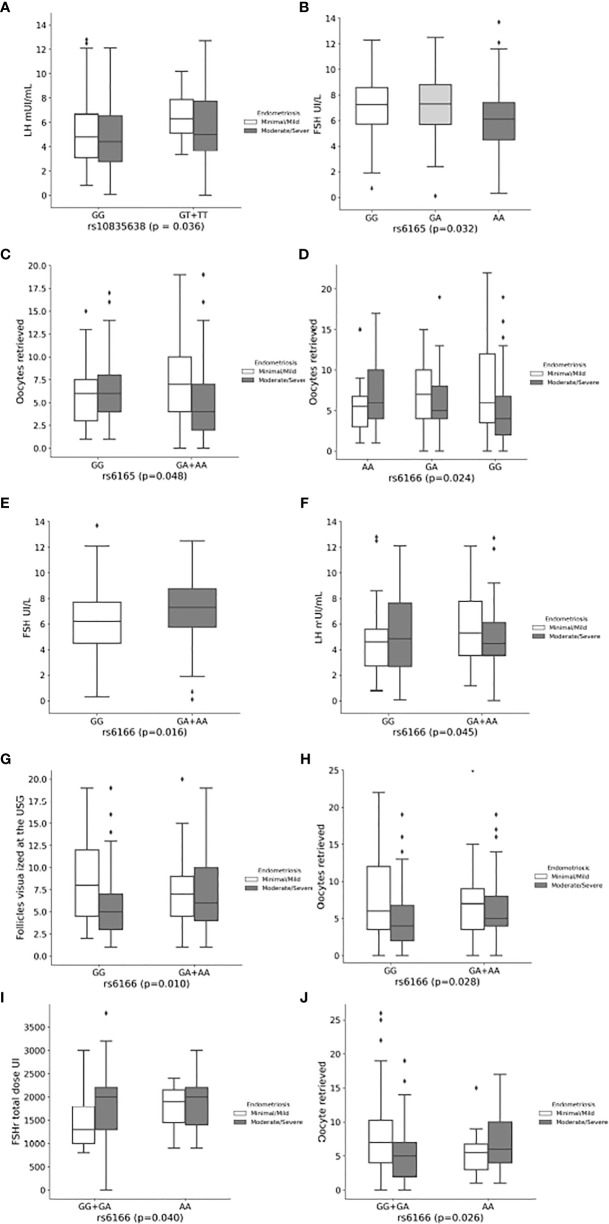
Effect of the *FSHB:*c.-211G>T, *FSHR:*c.919G>A and *FSHR:*c.2039G>A variants on hormone levels and reproductive outcomes of women with endometriosis. **(A)** Luteinizing hormone concentrations according to rs10835638 (*FSHB:*c.-211G>T) in overall and endometriosis´ stage. **(B)** Follicle-Stimulating Hormone concentrations according to rs6165 (*FSHR*:c.919G>A) in overall endometriosis. **(C)** Mean oocyte retrieved according to rs6165 (*FSHR:*c.919G>A). **(D)** Mean oocyte retrieved according to rs6166 genotypes (*FSHR*:c.2039G>A) in moderate/severe endometriosis. **(E)** Follicle-Stimulating Hormone concentrations according to rs6166 (*FSHR*:c.2039G>A) in in overall endometriosis. **(F)** Luteinizing hormone concentrations according to rs6166 (*FSHR:*c.2039G>A) in minimal/mild endometriosis. **(G)** Mean number of follicles visualized at the ultrasound according to rs6166 (*FSHR*:c.2039G>A) in moderate/severe endometriosis. **(H)** Mean number of oocytes retrieved according to rs6166 (*FSHR:*c.2039G>A) in moderate/severe endometriosis. **(I)** Mean dose of recombinant Follicle-Stimulating Hormone according to rs6166 (*FSHR*:c.2039G>A) in minimal/mild endometriosis. **(J)** Mean number of oocytes retrieved according to rs6166 (*FSHR*:c.2039G>A) in moderate/severe endometriosis.

The characteristics of women with overall endometriosis based on the presence of the *FSHR* and *FSHB* variants and their genetic models are shown in **Tables 3**–**5**, and in **Figure 1**. The characteristics of women with endometriosis according to the stage of the disease and the genetic models of the *FSHR* and *FSHB* variants are shown in **Supplementary Tables 1**–**3** and **Figure 1**.

**Table 3:** Characteristics of women with endometriosis according to dominant model of the *FSHB*:c.-211G>T variant.

Variables*	Dominant Model		
	*FSHB*:c.-211G>T		
	GG	GT+TT	p
**Endometriosis**			
N	164 (81.1%)	38 (18.8%)	–
LH (mUI/mL)	4.6 (4.1-5.1)	5.6 (4.6-7.5)	0.025
FSH (UI/L)	6.8 (6.5-7.3)	7.1 (6.2-8.4)	0.350
AFC	8 (6-9)	7 (6-9)	0.948
Days of COS	10 (10-11)	10 (10-11)	0.963
Total dose of rFSH (UI)	1800	1800	0.859
	(1600-2000)	(1400-2000)	
Follicles	6 (5-7)	6.5 (5-9)	0.865
Oocytes	6 (5-7)	5.5 (4-6)	0.385
MII	5 (4-6)	4 (4-5)	0.285
Embryos	3 (2-3)	3 (2-4)	0.814
Pregnancy rate/cycle	46	12	0.606
(n, %)	(33.8%)	(38.7%)	

*Qualitative variables were presented by absolute and relative frequency, and quantitative variables by median and 95% confidence interval. FSH, Follicle Stimulating Hormone; LH, Luteinizing Hormone; AFC, Antral Follicle Count; MII, Metaphase II oocytes.

**Table 4:** Characteristics of women with endometriosis according to genetic models and genotypes of the variants c.919G>A and c.2039G>A of the *FSHR* gene.

Variables*	Genetic Models									
	Additive				Dominant			Recessive		
***FSHR*:c.919G>A (rs6165:C>T, p.Ala307Thr)**										
	**GG**	**GA**	**AA**	**p**	**GG**	**GA+AA**	**p**	**GG+GA**	**AA**	**p**
N	56 (26.3%)	96 (45.1%)	61 (28.6%)		56 (26.3%)	157 (73.7%)		152 (71.4%)	61 (28.6%)	
LH (mUI/mL)	4.9 (4-5.9)	4.7 (4.3-5.7)	4.55 (3.7-5.66)	0.548	4.9 (4 - 5.9)	5.9 (4.7 - 4.3)	0.914	4.8 (4.3 - 5.7)	4.5 (3.7 - 5.7)	0.292
FSH (UI/L)	7.25 (6.4-7.8)	7.3 (6.6-7.9)	6.11 (5.3-6.9)	0.028^a^	7.2 (6.4 - 7.8)	7.8 (6.8 - 6.5)	0.423	7.3 (6.7 - 7.7)	6.1 (5.3 - 6.9)	0.008
AFC	6.5 (5-9)	7 (6-8)	8.5 (7-10)	0.253	6.5 (5 - 9)	9 (7.5 - 6)	0.771	7 (6 - 8)	8.5 (7 - 10)	0.107
Day of COS	10.5 (10-11)	10 (10-11)	10 (10-11)	0.689	10.5 (10 - 11)	11 (10 - 10)	0.402	10 (10 - 11)	10 (10 - 11)	0.893
Total dose of rFSH (UI)	2000 (1600-2200)	1800 (1400-1800)	1800 (1200-2000)	0.318	2000 (1600 - 2200)	2200 (1800 - 1600)	0.134	1800 (1800 - 2000)	1800 (1200 - 2000)	0.449
Follicles	6.5 (5-7)	6 (5-8)	6 (5-7)	0.984	6.5 (5 - 7)	7 (6 - 5)	0.863	6 (6 - 7)	6 (5 - 7)	0.94
Oocytes	6 (5-7)	5 (4-6)	6 (4-7)	0.598	6 (5 - 7)	7 (5 - 4)	0.443	6 (5 - 6)	6 (4 - 7)	0.749
MII	5 (4-6)	5 (4-6)	5 (4-7)	0.956	5 (4 - 6)	6 (5 - 4)	0.889	5 (4 - 5)	5 (4 - 7)	0.768
Embryos	3 (2-4)	3 (2-3)	3 (2-4)	0.739	3 (2 - 4)	4 (3 - 2)	0.956	3 (2 - 3)	3 (2 - 4)	0.492
Pregnancy rate/cycle (n, %)	20/51 (39.2%)	30/75 (40.0%)	15/52 (28.8%)	0.392	20/51 (39.2%)	45/127 (35.4%)	0.636	50/126 (39.7%)	15/52 (28.8%)	0.172
***FSHR:*c.2039G>A (rs6166:C>T, p.Ser680Asn)**										
	**GG**	**GA**	**AA**	**p**	**GG**	**GA+AA**	**p**	**GG+GA**	**AA**	**p**
N	73 (34.3%)	98 (46.0%)	42 (19.7%)		73 (35.7%)	140 (65.7%)		171 (80.3%)	42 (19.7%)	
LH (mUI/mL)	4.6 (3.7-5.7)	4.6 (4.1-5.6)	5.1 (4.3-6.1)	0.694	4.6 (3.7 - 5.7)	4.7 (4.3 - 5.7)	0.469	4.6 (4.1 - 5.2)	5.1 (4.3 - 6.1)	0.495
FSH (UI/L)	6.2 (5.4-6.9)	7.3 (6.7-7.7)	7.2 (6.2-8.1)	0.050	6.2 (5.4 - 6.9)	7.3 (6.7 - 7.7)	0.016	6.8 (6.6 - 7.3)	7.2 (6.2 - 8.1)	0.619
AFC	8 (7-10)	7 (6-8)	6 (5-9)	0.455	8 (7 - 10)	7 (6 - 8)	0.213	8 (6 - 8)	6 (5 - 9)	0.58
Day of COS	10 (10-11)	10 (10-11)	10 (10-11)	0.903	10 (10 - 11)	10 (10 - 11)	0.829	10 (10 - 11)	10 (10 - 11)	0.772
Total dose of rFSH (UI)	1800 (1300-2000)	1800 (1400-1800)	2000 (1600-2200)	0.275	1800 (1300 - 2000)	1800 (1800 - 2000)	0.564	1800 (1600 - 1800)	2000 (1600 - 2200)	0.108
Follicles	5 (4-7)	7 (6-8)	6 (5-9)	0.292	5 (4 - 7)	7 (6 - 8)	0.125	6 (5 - 7)	6 (5 - 9)	0.793
Oocytes	5 (3-6)	6 (5-7)	6 (5-8)	0.332	5 (3 - 6)	6 (5 - 7)	0.147	5 (5 - 6)	6 (5 - 8)	0.424
MII	4 (3-5)	5 (4-6)	5 (4-6)	0.609	4 (3 - 5)	5 (4 - 6)	0.341	5 (4 - 5)	5 (4 - 6)	0.937
Embryos	2.5 (2-4)	3 (2-4)	3 (2-4)	0.862	2.5 (2 - 4)	3 (2 - 3)	0.603	3 (2 - 3)	3 (2 - 4)	0.968
Pregnancy rate/cycle (n, %)	17/61 (27.9%)	34/80 (42.5%)	14/37 (37.8%)	0.199	17/61 (27.9%)	48/117 (41.0%)	0.083	51/141 (36.2%)	14/37 (37.8%)	0.851

*Qualitative variables were presented by absolute and relative frequency and quantitative variables by median and 95% confidence interval. FSH, Follicle Stimulating Hormone; LH, Luteinizing Hormone; AFC, Antral Follicle Count; COS, Controlled Ovarian Stimulation; rFSH, recombinant FSH; MII, Metaphase II oocytes. ^a^In the Dunn test GA genotype had significantly higher serum FSH levels compared to AA genotype (p=0.032).

**Table 5:** Analysis of the interaction model between the *FSHB*:c.-211G>T, *FSHR*:c.919G>A and *FSHR*:c.2039G>A variants in women with endometriosis, according to the MB-MDR model.

Variable	SNV 1	SNV 2	SNV 3	Betah	Wmax	Perm.P
FSH	rs6166	rs6165	—	—	5.004022	0.256
Follicles	rs6166	rs6165	—	0.1934041	14.60244	0.447
Oocytes	rs6166	rs6165	—	0.4977201	18.589954	0.285
MII	rs6166	rs6165	—		5.728913	0.73
Embryos	rs6166	rs6165	—	0.4778386	4.65169	0.749
FSH	rs10835638	rs6165	—	—	4.163284	0.311
Follicles	rs10835638	rs6165	—	0.1937734	6.328452	0.824
Oocytes	rs10835638	rs6165	—	0.3284823	3.915613	0.9
MII	rs10835638	rs6165	—	—	5.16851	0.739
Embryos	rs10835638	rs6165	—	0.1471549	3.024456	0.87
Follicles	rs10835638	rs6166	—	0.2545039	16.495389	0.359
Oocytes	rs10835638	rs6166	—	0.2238947	9.412196	0.645
MII	rs10835638	rs6166	—	0.1908889	7.678125	0.584
FSH	rs10835638	rs6166	rs6165	—	3.704604	0.62
Follicles	rs10835638	rs6166	rs6165	0.3103801	23.185487	0.491
Oocytes	rs10835638	rs6166	rs6165	0.2995511	21.226864	0.52
MII	rs10835638	rs6166	rs6165	0.1908889	13.438256	0.626
Embryos	rs10835638	rs6166	rs6165	0.4839351	4.762282	0.936

SNV, Single Nucleotide Variant. rs10835638: FSHB:c.-211G>T; rs6166: FSHR:c.2039G>A; rs:6165: FSHR:c.919G>A.

In relation to *FSHB*:c.-211G>T SNV (rs10835638), women carrying the T allele had significantly higher serum LH levels in overall (p=0.025) and minimal/mild endometriosis (p=0.036) in the dominant model (**Table 3** and **Supplementary Table 1**, respectively, and **Figure 1A**).

Considering the *FSHR*:c.919G>A SNV (rs6165, p.Ala307Thr), women with overall endometriosis carrying the GA genotype had significantly higher serum FSH levels than those with the AA genotype (p=0.032) in the additive model (**Table 4** and **Figure 1B**). The GG+GA genotypes were also associated with higher serum FSH levels compared to AA genotype in the recessive model (**Table 4**), the same was observed in minimal/mild disease (**Supplementary Table 2**). In addition, women with moderate/severe endometriosis carrying the A allele had a significantly lower number of oocytes retrieved (p=0.048) in the dominant model (**Supplementary Table 2** and **Figure 1C**).

Regarding the *FSHR:*c.2039G>A SNV (rs6166, p.Ser680Asn), women with moderate/severe endometriosis carrying the AA genotype had a significantly higher number of oocytes retrieved than those carrying the GG genotype (p=0.024) in the additive model (**Supplementary Table 3** and **Figure 1D**). Moreover, in the dominant model, women carrying the A allele showed significantly higher FSH levels (p=0.016) in overall endometriosis (**Table 4** and **Figure 1E**), while women with minimal/mild endometriosis showed significantly higher LH levels (p=0.045) (**Supplementary Table 3** and **Figure 1F**), and women with moderate/severe endometriosis showed a statistically greater number of follicles visualized by ultrasound and a higher number of oocytes retrieved (p=0.010 and p=0.028, respectively) (**Supplementary Table 3** and **Figures 1G, H**). In the recessive model, women with minimal/mild endometriosis carrying the AA genotype used a higher dose of recombinant FSH (rFSH) (p=0.040), while those with moderate/severe disease showed a greater number of oocytes retrieved (p=0.026) (**Supplementary Table 3** and **Figures 1I, J**).

Analysis of gene interactions among the three variants, *FSHB:*c.-211G>T, *FSHR*:c.2039G>A, and *FSHR*:c.919G>A, according to the MB-MDR model, showed that the combination of these variants did not interfere with the hormonal or reproductive characteristics of the women studied (**Table 5**).

## Discussion

To the best of our knowledge, this is the first study to evaluate the effect of the variants of *FSHB* and *FSHR* on the hormonal profile and reproductive outcomes of infertile women with endometriosis. The A allele of the *FSHR*:c.919G>A SNV was associated with a lower number of oocytes retrieved in women with moderate/severe endometriosis and the GA genotype was associated with higher FSH levels in overall endometriosis. The A allele of the *FSHR*:c.2039G>A SNV was associated with higher FSH levels in overall endometriosis and higher LH levels in minimal/mild disease, while in moderate/severe endometriosis it was associated with a greater number of follicles visualized by ultrasound and a higher number of retrieved oocytes. The AA genotype was associated with a higher dose of rFSH to perform COS in minimal/mild disease and a greater number of oocytes retrieved in moderate/severe endometriosis. The T allele of the *FSHB*:c.-211G>T SNV was associated with higher serum LH levels in women with overall and minimal/mild endometriosis. Taken together, the interactions among the three variants studied showed no association.

The allelic frequencies of the SNVs studied were similar to those found in the Brazilian Online Archive of Mutations (ABraOM), the Project “1000 genomes”, and Trans-Omics for Precision Medicine (TOPMed), representative of the global population. The minor allele frequency (MAF) of the *FSHB* (rs10835638:G>T) SNV was 9.9% in the present study, 10.8% in the ABraOM, 8.4% in the 1000 genomes, and 11.2% in TOPMed. Regarding the *FSHR* (rs6165: C>T) SNV, MAF was 48.8% in the present study, 49.3%, 49.2%, and 47.8% in ABraOM, 1000 genomes, and TOPMed, respectively. The MAF of the *FSHR* (rs6166:C>T) SNV was 42.7% in our study, 51.6% in the ABraOM, 40.7% in the 1000 genomes, and 42.5% in TOPMed.

Previous studies have shown an association between the T allele of the *FSHB*:c.-211G>T and lower levels of FSH and LH (13, 17–22), longer intervals between menstrual cycles (20), idiopathic infertility (29), and IVF outcomes (7, 13, 17–22). Moreover, in a study from the UK Biobank, the T allele was associated with longer menstrual cycles and increased age at menopause. Moreover, although it showed detrimental effects on fertility, it was protective against endometriosis (21). However, no study has evaluated the effect of this variant on the reproductive outcomes of women with endometriosis.

Recently, our group (7) studied the effect of *FSHB*:c.-211G>T SNV on the reproductive outcomes in 140 Brazilian women with infertility mainly caused by males or tuboperitoneal abnormalities (except endometriosis). Women carrying the GT genotype had poorer responses to COS when compared to those with the GG genotype (47.4% *vs*. 26.5%), significantly higher LH levels (3.1 IU/mL *vs*. 2.4 IU/mL), lower AFC (8.0 *vs*. 10.0), oocytes retrieved (3.0 *vs*. 5.0), MII (3.0 *vs*. 4.0), and embryos (2.0 *vs*. 3.0). Despite these findings, no difference was observed in pregnancy rates. In the present study, the T variant allele was significantly associated only with higher LH levels in overall endometriosis (5.6 mUI/mL *vs* 4.6 mUI/mL) and minimal/mild disease (6.3 mUI/mL *vs*. 4.8 mUI/mL).

*FSHR*:c.919G>A and *FSHR*:c.2039G>A were previously associated with variability in serum FSH levels and reproductive outcomes in different populations (11–13, 15, 30–34). These SNVs are located in the coding region of the *FSHR* gene and have been well characterized (13). The SNV known as Serine680 (*FSHR*:c.2039G>A, rs6166:C>T, p.Ser680Asn) variant is a missense mutation that causes the replacement of asparagine (Asn) for serine (Ser) at the 680 position, which is located in the intracellular domain of the FSHR protein, introducing a potential phosphorylation site. The rs6165 SNV (*FSHR:*c.919G>A, rs6165:C>T, p.Ala307Thr) is also a missense mutation that replaces threonine (Thr) with alanine (Ala), 6165), a non-polar hydrophobic amino acid, which removes a potential O-linked glycosylation site (13, 35).

The *FSHR*:c.2039G>A shows a high degree of linkage disequilibrium with *FSHR*:c.919G>A many ethnic groups (36), segments of the genome in which a given combination of alleles or genetic markers is inherited coordinately. In the present study, these two *FSHR* variants are not in linkage disequilibrium. Different allele frequencies between populations arise from different genetic ancestors, so the racial origins of the studied population may be responsible for the divergent results. The Brazilian population has contributions from three main parental groups (Amerindian, European and African), and this heterogeneity can produce allele frequencies different from those presented by non-mixed populations (37–39).

Recently, a systematic review followed by a meta-analysis was performed by Alviggi et al. (2018) (16), aiming to define the impact of specific genotype profiles of FSH, LH, and their receptors (*FSHR*, *LHR*, and *LHCGR*) on ovarian stimulation outcomes. The results suggest that more oocytes were retrieved from *FSHR* (rs6165) AA homozygotes, and stimulation duration was shorter in *FSHR* (rs6165) AA homozygotes. In vitro studies using human granulosa cells showed that GG carriers of the *FSHR*:c.2039G>A genotype have greater resistance to FSH than AA carriers (35, 40). In the present study, the A allele of the *FSHR*:c.919G>A SNV was associated with a lower number of oocytes retrieved in moderate/severe endometriosis, and no association was found between genotypes or alleles of this SNV and the duration of COS. Concerning *FSHR*:c.2039G>A SNV, AA genotype was associated with a higher amount of rFSH for COS in women with minimal/mild disease and a greater number of oocytes retrieved in women with moderate/severe endometriosis.

Anagnostou et al. (2020) (11) evaluated the impact of the rs6166 variant of the *FSHR* gene and the rs10835638 variant of the *FSHB* gene in a Greek population of women undergoing IVF/ICSI. The results showed that the *FSHB* polymorphism appears to be quite rare in the infertile Greek population. In addition, the two polymorphisms separately showed statistically significant differences in LH levels, and women with 2-3 combined polymorphic alleles needed more days of stimulation, but there were no differences in pregnancy rates. In the present study, the TT genotype of the *FSHB* SNV was also rare and was found in only two women. The T allele of the *FSHB*:c.-211G>T SNV and the A allele of the *FSHR*:c.2039G>A SNV were associated with higher LH levels. However, the combined alleles of the SNVs showed no association.

Polyzos et al. (2021) (41) conducted a multicenter multinational prospective study, including 368 patients from Vietnam, Belgium, and Spain (168 from Europe and 200 from Asia), to verify whether the presence of SNVs in the *FSHR* gene (rs6165, rs6166, and rs1394205) and/or *FSHB* gene (rs10835638) influence ovarian response in predicted normal responders treated with rFSH. They found that the presence of *FSHR* SNPs has a statistically significant impact on ovarian response, although this effect is of minimal clinical relevance in predicted normal responders treated with a fixed dose of 150 IU rFSH. In the present study, the A allele of the *FSHR*:c.919G>A SNV was associated with a lower number of oocytes only in women with moderate/severe disease, while the AA genotype of the *FSHR*, c.2039G>A SNV, was associated with a lower amount of rFSH for COS in women with minimal/mild disease and a greater number of oocytes retrieved in women with moderate/severe endometriosis.

Interestingly, the findings were quite different considering the effect of the *FSHR*:c.2039G>A variant on endometriosis stage. This SNV was found to affect LH levels and total amount of rFSH in those with minimal/mild disease, and number of follicles and number of oocytes retrieved in those with moderate/severe endometriosis. Considering this, we may speculate that this subpopulation of infertile women may obtain best results during COS when stimulated with higher dosage of gonadotropins, especially in terms of number of oocytes retrieved. Besides this, a potential consideration for the clinical practice is that our data may improve the patient counseling regarding the results of COS in this subpopulation.

Although we acknowledge that this type of genetic investigation is currently limited to research activities, and it may be not cost-effective in the clinical practice, we take the opportunity to solicit further analysis to assess whether infertile women with endometriosis carrying *FSHR:c*.2039G>A SNV should undergo different COS strategies according to the stage of the disease in order to obtain the best outcomes.

Concerning the study limitations, our study was performed in relatively young women with normal ovarian reserve to eliminate biases related to age-related fertility decline. In addition, we found only two women with variant homozygote genotype of the *FSHB*:c.-211G>T SNV, which limited the analysis. Moreover, only infertile women with endometriosis were included.

## Conclusions

In conclusion, the findings of the present study suggest that, when examined individually, the presence of the variant T allele in the *FSHB*:c.-211G>T SNV affected LH levels in women with overall endometriosis and minimal/mild disease. *FSHR*:c.919G>A SNV affected FSH levels in women with overall endometriosis and the number of oocytes retrieved in those with moderate/severe endometriosis. Moreover, the *FSHR:*c.2039G>A SNV affected FSH levels in women with overall endometriosis, LH levels and total amount of rFSH in those with minimal/mild disease, and number of follicles and number of oocytes retrieved in those with moderate/severe endometriosis. No effect on hormone profile or reproductive outcomes was observed when the genotypes were combined.

Taken together, our findings show that it is of paramount importance to investigate more genes and their variants in different ethnicities and identify the causes of infertility to understand the mechanisms by which they interact and affect the hormones and the response to COS.

## Data Availability Statement

The datasets used in this study are available at 10.17632/2yhwkbw74m.1.

## Ethics Statement 

The study was approved by the Research Ethics Committee of the “Faculdade de Medicina do ABC/Centro Universitário FMABC” (approve code #039/2011 and date of approval 04/27/2011, and CAEE CAAE 64167716.9.1001.0082 and date of approval 05/19/2017). Each patient enrolled in this study signed an informed consent for all the procedures and to allow data and biological sample collection and analysis for research purposes. The patients/participants provided their written informed consent to participate in this study.

## Author Contributions

Conceptualization: BB. Methodology: BB, DC, and CB. Software: CT and EM. Validation: BB, FL, and CT. Formal analysis: BB, CT, and EM. Investigation: FL, CT, and CP. Resources: BB, DC, and CB. Data curation: BB, CT, and EM. Writing – original draft preparation: BB and FL. Writing – review and editing: BB, EM, and AL. Supervision: BB. Visualization: BB and EM. Project administration: BB. Funding acquisition: BB. All authors critically reviewed the manuscript and approved the final version of the manuscript. All authors contributed to the article and approved the submitted version.

## Funding

This work was supported by The São Paulo Research Foundation-FAPESP research grant #2016/25953-9.

## Conflict of Interest

The authors declare that the research was conducted in the absence of any commercial or financial relationships that could be construed as a potential conflict of interest.

## Publisher’s Note

All claims expressed in this article are solely those of the authors and do not necessarily represent those of their affiliated organizations, or those of the publisher, the editors and the reviewers. Any product that may be evaluated in this article, or claim that may be made by its manufacturer, is not guaranteed or endorsed by the publisher.

## Abbreviations

AFC, Antral follicle counting; ART, Assisted Reproductive Treatment; COS, Controlled ovarian stimulation; FSH, Follicle-stimulating hormone; FSHB, hormone-specific β-chain; FSHR, Follicle-stimulating hormone receptor; HWE, Hardy-Weinberg equilibrium; IVF, In vitro fertilization; LH, Luteinizing Hormone; MB-MDR, Multifactorial dimensionality reduction; rFSH, recombinant Follicle-stimulating hormone; SNV, Single Nucleotide Variants.

## References

[1] Laganà AS Garzon S Götte M Viganò P Franchi M Ghezzi F. The Pathogenesis of Endometriosis: Molecular and Cell Biology Insights. Int J Mol Sci (2019) 20(22):5615. doi: 10.3390/ijms20225615 PMC6888544 31717614

[2] Šalamun V Verdenik I Laganà AS Vrtačnik-Bokal E. Should We Consider Integrated Approach for Endometriosis-Associated Infertility as Gold Standard Management? Rationale and Results From a Large Cohort Analysis. Arch Gynecol Obstet (2018) 297(3):613–21. doi: 10.1007/s00404-017-4633-0 29274003

[3] Taylor HS Kotlyar AM Flores VA. Endometriosis is a Chronic Systemic Disease: Clinical Challenges and Novel Innovations. Lancet (2021) 397(10276):839–52. doi: 10.1016/S0140-6736(21)00389-5 33640070

[4] Gu BH Park JM Baek KH. Genetic Variations of Follicle Stimulating Hormone Receptor are Associated With Polycystic Ovary Syndrome. Int J Mol Med (2010) 26(1):107–12. doi: 10.3892/ijmm_00000441 20514429

[5] Fan QR Hendrickson WA. Structure of Human Follicle-Stimulating Hormone in Complex With its Receptor. Nature (2005) 433(7023):269–77. doi: 10.1038/nature03206 PMC5514322 15662415

[6] Nagirnaja L Rull K Uusküla L Hallast P Grigorova M Laan M. Genomics and Genetics of Gonadotropin Beta-Subunit Genes: Unique FSHB and Duplicated LHB/CGB Loci. Mol Cell Endocrinol (2010) 329(1-2):4–16. doi: 10.1016/j.mce.2010.04.024 20488225 PMC2954307

[7] Trevisan CM de Oliveira R Christofolini DM Barbosa CP Bianco B. Effects of a Polymorphism in the Promoter Region of the Follicle-Stimulating Hormone Subunit Beta (FSHB) Gene on Female Reproductive Outcomes. Genet Test Mol Biomarkers (2019) 23(1):39–44. doi: 10.1089/gtmb.2018.0182 30585745

[8] Altmäe S Hovatta O Stavreus-Evers A Salumets A. Genetic Predictors of Controlled Ovarian Hyperstimulation: Where do We Stand Today? Hum Reprod Update (2011) 17(6):813–28. doi: 10.1093/humupd/dmr034 21862569

[9] Roque M Bianco B Christofolini DM Barchi Cordts E Vilarino F Carvalho W. Pharmacogenetic Algorithm for Individualized Controlled Ovarian Stimulation in Assisted Reproductive Technology Cycles. Panminerva Med (2019) 61(1):76–81. doi: 10.23736/S0031-0808.18.03496-1 29916218

[10] Tafazoli A Wołczyński S Wawrusiewicz-Kurylonek N Esmaeili SA Miltyk W. Pharmacogenomic Biomarkers of Follicle-Stimulating Hormone Receptor Malfunction in Females With Impaired Ovarian Response-A Genetic Survey. J Clin Med (2021) 10(2):170. doi: 10.3390/jcm10020170 33561079 PMC7825139

[11] Anagnostou E Kafkoutsou A Mavrogianni D Domali E Dimitroulia E Mathiopoulos D. Individual and Combined Assessment of Ser680Asn FSH Receptor and Fshβ -211 G>T Gene Polymorphisms in Ovarian Response in IVF/ICSI Program. Curr Pharm Biotechnol (2020) 22(14):1857–65. doi: 10.2174/1389201021666201029153518 33121406

[12] Trevisan CM Peluso C Cordts EB de Oliveira R Christofolini DM Barbosa CP. Ala307Thr and Asn680Ser Polymorphisms of FSHR Gene in Human Reproduction Outcomes. Cell Physiol Biochem (2014) 34(5):1527–35. doi: 10.1159/000366356 25322982

[13] Simoni M Casarini L. Mechanisms in Endocrinology: Genetics of FSH Action: A 2014-and-Beyond View. Eur J Endocrinol (2014) 170(3):R91–107. doi: 10.1530/EJE-13-0624 24288354

[14] Pabalan N Trevisan CM Peluso C Jarjanazi H Christofolini DM Barbosa CP. Evaluating Influence of the Genotypes in the Follicle-Stimulating Hormone Receptor (FSHR) Ser680Asn (Rs6166) Polymorphism on Poor and Hyper-Responders to Ovarian Stimulation: A Meta-Analysis. J Ovarian Res (2014) 7:285. doi: 10.1186/s13048-014-0122-2 25526787 PMC4279698

[15] Tang H Yan Y Wang T Zhang T Shi W Fan R. Effect of Follicle-Stimulating Hormone Receptor Asn680Ser Polymorphism on the Outcomes of Controlled Ovarian Hyperstimulation: An Updated Meta-Analysis of 16 Cohort Studies. J Assist Reprod Genet (2015) 32(12):1801–10. doi: 10.1007/s10815-015-0600-5 PMC4681732 26481502

[16] Alviggi C Conforti A Santi D Esteves SC Andersen CY Humaidan P. Clinical Relevance of Genetic Variants of Gonadotrophins and Their Receptors in Controlled Ovarian Stimulation: A Systematic Review and Meta-Analysis. Hum Reprod Update (2018) 24(5):599–614. doi: 10.1093/humupd/dmy019 29924306

[17] La Marca A Papaleo E Alviggi C Ruvolo G De Placido G Candiani M. The Combination of Genetic Variants of the FSHB and FSHR Genes Affects Serum FSH in Women of Reproductive Age. Hum Reprod (2013) 28(5):1369–74. doi: 10.1093/humrep/det061 23504007

[18] Day FR Hinds DA Tung JY Stolk L Styrkarsdottir U Saxena R. Causal Mechanisms and Balancing Selection Inferred From Genetic Associations With Polycystic Ovary Syndrome. Nat Commun (2015) 6:8464. doi: 10.1038/ncomms9464 26416764 PMC4598835

[19] Hayes MG Urbanek M Ehrmann DA Armstrong LL Lee JY Sisk R. Genome-Wide Association of Polycystic Ovary Syndrome Implicates Alterations in Gonadotropin Secretion in European Ancestry Populations. Nat Commun (2015) 6:7502. doi: 10.1038/ncomms8502 26284813 PMC4557132

[20] Laisk-Podar T Kaart T Peters M Salumets A. Genetic Variants Associated With Female Reproductive Ageing–Potential Markers for Assessing Ovarian Function and Ovarian Stimulation Outcome. Reprod BioMed Online (2015) 31(2):199–209. doi: 10.1016/j.rbmo.2015.05.001 26099445

[21] Ruth KS Beaumont RN Tyrrell J Jones SE Tuke MA Yaghootkar H. Genetic Evidence That Lower Circulating FSH Levels Lengthen Menstrual Cycle, Increase Age at Menopause and Impact Female Reproductive Health. Hum Reprod (2016) 31(2):473–81. doi: 10.1093/humrep/dev318 PMC4716809 26732621

[22] Ruth KS Campbell PJ Chew S Lim EM Hadlow N Stuckey BG. Genome-Wide Association Study With 1000 Genomes Imputation Identifies Signals for Nine Sex Hormone-Related Phenotypes. Eur J Hum Genet (2016) 24(2):284–90. doi: 10.1038/ejhg.2015.102 PMC4564946 26014426

[23] Classification of Endometriosis of the American Society for Reproductive Medicine. Revised American Society for Reproductive Medicine Classification of Endometriosis: 1996. Fertil Steril (1997) 67(5):817–21. doi: 10.1016/s0015-0282(97)81391-x 9130884

[24] The American College of Obstetricians and Gynecologists to women fertility investigation. Infertility Workup for the Women's Health Specialist: ACOG Committee Opinion, Number 781. Obstet Gynecol (2019) 133(6):e377–84. doi: 10.1097/AOG.0000000000003271 31135764

[25] American Society for Reproductive Medicine to Diagnostic Evaluation of the Infertile Female. Practice Committee of the American Society for Reproductive Medicine. Diagnostic Evaluation of the Infertile Female: A Committee Opinion. Fertil Steril (2015) 103(6):e44–50. doi: 10.1016/j.fertnstert.2015.03.019 25936238

[26] Broekmans FJ de Ziegler D Howles CM Gougeon A Trew G Olivennes F. The Antral Follicle Count: Practical Recommendations for Better Standardization. Fertil Steril (2010) 94(3):1044–51. doi: 10.1016/j.fertnstert.2009.04.040 19589513

[27] R Core Team. R: A Language and Environment for Statistical Computing. R Foundation for Statistical Computing. Vienna, Austria: R Foundation for Statistical Computing (2018).

[28] Calle ML Urrea V Malats N Van Steen K. Mbmdr: An R Package for Exploring Gene-Gene Interactions Associated With Binary or Quantitative Traits. Bioinformatics (2010) 26(17):2198–9. doi: 10.1093/bioinformatics/btq352 20595460

[29] Rull K Grigorova M Ehrenberg A Vaas P Sekavin A Nõmmemees D. FSHB -211 G>T Is a Major Genetic Modulator of Reproductive Physiology and Health in Childbearing Age Women. Hum Reprod (2018) 33(5):954–66. doi: 10.1093/humrep/dey057 29617818

[30] Allegra A Marino A Raimondo S Maiorana A Gullo S Scaglione P. The Carriers of the a/G-G/G Allelic Combination of the C.2039 a>G and C.-29 G>A FSH Receptor Polymorphisms Retrieve the Highest Number of Oocytes in IVF/ICSI Cycles. J Assist Reprod Genet (2017) 34(2):263–73. doi: 10.1007/s10815-016-0835-9 PMC5306403 27817039

[31] García-Jiménez G Zariñán T Rodríguez-Valentín R Mejía-Domínguez NR Gutiérrez-Sagal R Hernández-Montes G. Frequency of the T307A, N680S, and -29G>A Single-Nucleotide Polymorphisms in the Follicle-Stimulating Hormone Receptor in Mexican Subjects of Hispanic Ancestry. Reprod Biol Endocrinol (2018) 16(1):100. doi: 10.1186/s12958-018-0420-4 30340493 PMC6195735

[32] König TE van der Lee J Schats R Lambalk CB. The Relationship Between FSH Receptor Polymorphism Status and IVF Cycle Outcome: A Retrospective Observational Study. Reprod BioMed Online (2019) 39(2):231–40. doi: 10.1016/j.rbmo.2019.05.018 31279715

[33] Paschalidou C Anagnostou E Mavrogianni D Raouasnte R Klimis N Drakakis P. The Effects of Follicle-Stimulating Hormone Receptor (FSHR) -29 and Ser680Asn Polymorphisms in IVF/ICSI. Horm Mol Biol Clin Investig (2020) 41(2):1–11. doi: 10.1515/hmbci-2019-0058 32114522

[34] Ahmed I Abdelateef S Abdel-Lah MA Amor H Hammadeh ME. Association Between FSHR and ESR1 Gene Variants and Ovarian Response to Gonadotropin in Egyptian Women Undergoing ICSI Treatment. Reprod Biol (2021) 21(2):100499. doi: 10.1016/j.repbio.2021.100499 33740738

[35] Conforti A Vaiarelli A Cimadomo D Bagnulo F Peluso S Carbone L. Pharmacogenetics of FSH Action in the Female. Front Endocrinol (Lausanne) (2019) 10:398. doi: 10.3389/fendo.2019.00398 31293516 PMC6606727

[36] Perez Mayorga M Gromoll J Behre HM Gassner C Nieschlag E Simoni M. Ovarian Response to Follicle-Stimulating Hormone (FSH) Stimulation Depends on the FSH Receptor Genotype. J Clin Endocrinol Metab (2000) 85(9):3365–9. doi: 10.1210/jc.85.9.3365 10999835

[37] Wang S Ray N Rojas W Parra MV Bedoya G Gallo C. Geographic Patterns of Genome Admixture in Latin American Mestizos. PloS Genet (2008) 4(3):e1000037. doi: 10.1371/journal.pgen.1000037 18369456 PMC2265669

[38] Salzano FM Sans M. Interethnic Admixture and the Evolution of Latin American Populations. Genet Mol Biol (2014) 37(1 Suppl):151–70. doi: 10.1590/S1415-47572014000200003 PMC3983580 24764751

[39] Giolo SR Soler JM Greenway SC Almeida MA de Andrade M Seidman JG. Brazilian Urban Population Genetic Structure Reveals a High Degree of Admixture. Eur J Hum Genet (2012) 20(1):111–6. doi: 10.1038/ejhg.2011.144 PMC3234512 21863058

[40] Casarini L Moriondo V Marino M Adversi F Capodanno F Grisolia C. FSHR Polymorphism P.N680S Mediates Different Responses to FSH In Vitro. Mol Cell Endocrinol (2014) 393(1-2):83–91. doi: 10.1016/j.mce.2014.06.013 24970684

[41] Polyzos NP Neves AR Drakopoulos P Spits C Alvaro Mercadal B Garcia S. The Effect of Polymorphisms in FSHR and FSHB Genes on Ovarian Response: A Prospective Multicenter Multinational Study in Europe and Asia. Hum Reprod (2021) 36(6):1711–21. doi: 10.1093/humrep/deab068 33889959

