# Supplementary material for: Effects of FSHR and FSHB Variants on Hormonal Profile and Reproductive Outcomes of Infertile Women With Endometriosis
Source: Front Endocrinol (Lausanne). 2021 Sep 30;12:760616. doi: 10.3389/fendo.2021.760616 (PMC8514996; doi:10.3389/fendo.2021.760616)
Supplement: Supplementary file 1 [file DataSheet_1.docx]

**Supplementary Table 1.** Characteristics of women with endometriosis according to the stage of the disease and regarding the dominant model of the *FSHB:*c.-211G>T variant.

| **Variables*** | **Dominant Model**  ***FSHB*:c.-211G>T (rs10835638)** | | |  |
| --- | --- | --- | --- | --- |
|  | **GG** | **GT+TT** | **p** |  |
| **Minimal/Mild Endometriosis** | | | |  |
| N | 62 (79.5%) | 16 (20.5%) | -- |  |
| LH (mUI/mL) | 4.8 (3.9-5.7) | 6.3 (5.1-7.9) | **0.036** |  |
| FSH (UI/L) | 6.83 (6.0-7.4) | 6.6 (5.1-8.4) | 0.748 |  |
| AFC | 8 (7-9) | 9 (7-13) | 0.243 |  |
| Day of COS | 10 (9-11) | 10 (9-12) | 0.889 |  |
| Total dose of rFSH (UI) | 1550  (1200-1800) | 1400  (900-2000) | 0.501 |  |
| Follicles | 7 (6-9) | 8.5 (5-9) | 0.970 |  |
| Oocytes | 7 (6-8) | 6 (3-9) | 0.780 |  |
| MII | 6 (4-7) | 5 (3-8) | 0.471 |  |
| Embryos | 3 (2-4) | 3.5 (1-5) | 0.815 |  |
| Pregnancy rate/cycle  (n, %) | 19/50  (38.0%) | 6/12  (50%) | 0.665 |  |
| **Moderate/Severe Endometriosis** | | | | |
| N | 102 (82.3%) | 22 (17.7%) | -- |  |
| LH (mUI/mL) | 4.4 (3.9-5.6) | 5 (3.7-7.7) | 0.276 |  |
| FSH (UI/L) | 6.8 (6.2-7.7) | 7.3 (6.2-9.9) | 0.154 |  |
| AFC | 6 (5-9) | 6 (4-8) | 0.688 |  |
| Day of COS | 10 (10-11) | 11 (9-12) | 0.763 |  |
| Total dose of rFSH (UI) | 2000  (1800-2000) | 2000  (1800-2200) | 0.356 |  |
| Follicles | 6 (5-7) | 6 (4-9) | 0.880 |  |
| Oocytes | 5 (4-6) | 4 (3-6) | 0.290 |  |
| MII | 5 (4-5) | 4 (3-6) | 0.340 |  |
| Embryos | 3 (2-4) | 2 (1-4) | 0.528 |  |
| Pregnancy rate/cycle  (n, %) | 27/86  (31.4%) | 6/19  (31.6%) | 0.988 |  |

*Qualitative variables were presented by absolute and relative frequency and quantitative variables by median and 95% confidence interval. FSH: Follicle Stimulating Hormone; LH: Luteinizing Hormone; AFC: Antral Follicle Count; MII: Metaphase II oocytes.

**Supplementary Table 2.** Characteristics of women with endometriosis according to the stage of the disease and regarding the genetic models of the *FSHR*:c.919G>A variant.

| **Variables** | **Genetics Models of the  *FSHR*:c.919G>A (rs6165, p.Ala307Thr)** | | | | | | | | | | | |
| --- | --- | --- | --- | --- | --- | --- | --- | --- | --- | --- | --- | --- |
|  | **Additive** | | | |  | **Dominant** | | |  | **Recessive** | | |
|  | **GG** | **GA** | **AA** | **p** |  | **GG** | **GA+AA** | **p** |  | **GG+GA** | **AA** | **p** |
| **Minimal/Mild Endometriosis** | | | | | | | | | | | | |
| N | 19 (23.2%) | 37 (45.1%) | 26 (31.7%) |  |  | 56 (26.3%) | 157 (73.7%) |  |  | 152 (71.4%) | 61 (28.6%) |  |
| LH (mUI/mL) | 5.0 (3.5-7.4) | 5.6 (4.3-7.3) | 4.6 (2.7-5.5) | 0.153 |  | 4.9 (4 - 5.9) | 5.9 (4.7 - 4.3) | 0.914 |  | 4.8 (4.3 - 5.7) | 4.5 (3.7 - 5.7) | 0.292 |
| FSH (UI/L) | 7.4 (5.1-9.4) | 7.4 (6.3-8.4) | 6.6 (5.4-6.9) | 0.185 |  | 7.2 (6.4 - 7.8) | 7.8 (6.8 - 6.5) | 0.423 |  | 7.4 (6.4 -8.0) | 6.6 (5.4– 6.9) | 0.079 |
| AFC | 7 (4-11) | 8 (6-9) | 9 (8-10) | 0.301 |  | 6.5 (5 - 9) | 9 (7.5 - 6) | 0.771 |  | 7 (6 - 8) | 8.5 (7 - 10) | 0.107 |
| Day of COS | 10 (9-12) | 10 (9-11) | 9.5 (9-12) | 0.591 |  | 10.5 (10 - 11) | 11 (10 - 10) | 0.402 |  | 10 (10 - 11) | 10 (10 - 11) | 0.893 |
| Total dose of rFSH (UI) | 1800  (1000-2200) | 1400  (1100-1800) | 1200  (900-1800) | 0.304 |  | 2000  (1600 - 2200) | 2200  (1800 - 1600) | 0.134 |  | 1800  (1800 - 2000) | 1800  (1200 - 2000) | 0.449 |
| Follicles | 6 (4-9) | 7 (6-9) | 8.5 (5-11) | 0.461 |  | 6.5 (5 - 7) | 7 (6 - 5) | 0.863 |  | 6 (6 - 7) | 6 (5 - 7) | 0.94 |
| Oocytes | 6 (3-8) | 7 (5-9) | 6 (5-11) | 0.545 |  | 6 (5 - 7) | 7 (5 - 4) | 0.443 |  | 6 (5 - 6) | 6 (4 - 7) | 0.749 |
| MII | 4 (2-8) | 5 (5-7) | 6 (3-9) | 0.613 |  | 5 (4 - 6) | 6 (5 - 4) | 0.889 |  | 5 (4 - 5) | 5 (4 - 7) | 0.768 |
| Embryos | 2 (1-4) | 3 (2-5) | 3 (2-6) | 0.356 |  | 3 (2 - 4) | 4 (3 - 2) | 0.956 |  | 3 (2 - 3) | 3 (2 - 4) | 0.492 |
| Pregnancy rate/cycle  (n, %) | 7/16  (43.8%) | 14/29  (48.3%) | 7/21  (33.3%) | 0.569 |  | 20/51  (39.2%) | 45/127 (35.4%) | 0.636 |  | 50/126 (39.7%) | 15/52 (28.8%) | 0.172 |
| **Moderate/Severe Endometriosis** | | | | | | | | | | | | |
| N | 37 (28.2%) | 59 (45.0%) | 35 (26.7%) | -- |  | 37 (28.2%) | 94 (71.8%) |  |  | 96 (73.3%) | 35 (26.7%) | -- |
| LH (mUI/mL) | 4.8 (3.6-6.1) | 4.5 (3.9-5.7) | 4.4 (3.5-6.5) | 0.999 |  | 4.8 (3.6-6.1) | 4.5 (3.9-5.7) | 0.973 |  | 4.6 (4 - 5.6) | 4.4 (3.5 - 6.5) | 0.969 |
| FSH (UI/L) | 7.2 (6.2-8.2) | 7.3 (6.6-8) | 6.1 (4.4-7.6) | 0.165 |  | 7.2 (6.2-8.2) | 6.7 (6.2-7.7) | 0.443 |  | 7.2 (6.6 - 7.8) | 6.1 (4.4 - 7.6) | 0.058 |
| AFC | 6.5 (5-9) | 6 (4-8) | 7 (3-10) | 0.454 |  | 6.5 (5-9) | 6 (5-7) | 0.382 |  | 6 (5 - 8) | 7 (3 - 10) | 0.637 |
| Day of COS | 11 (10-11) | 10 (10-12) | 10.5 (10-11) | 0.792 |  | 11 (10-11) | 10 (10-11) | 0.699 |  | 10.5 (10 - 11) | 10.5 (10 - 11) | 0.708 |
| Total dose of rFSH (UI) | 2000  (1600-2200) | 1800  (1800-2000) | 2000  (1300-2200) | 0.716 |  | 2000  (1600-2200) | 2000  (1800-2000) | 0.454 |  | 2000  (1800 - 2000) | 2000  (1300 - 2200) | 0.986 |
| Follicles | 7 (5-8) | 5 (4-7) | 5 (4-7) | 0.403 |  | 7 (5-8) | 5 (4-6) | 0.262 |  | 6 (5 - 7) | 5 (4 - 7) | 0.266 |
| Oocytes | 6 (5-7) | 4 (4-6) | 5 (3-7) | 0.133 |  | 6 (5-7) | 4 (4-5) | **0.048** |  | 5 (4 - 6) | 5 (3 - 7) | 0.674 |
| MII | 5 (4-6) | 4 (3-5) | 4.5 (2-7) | 0.704 |  | 5 (4-6) | 4 (4-5) | 0.405 |  | 5 (4 - 5) | 4.5 (2 - 7) | 0.791 |
| Embryos | 3 (2-4) | 2 (1-3) | 3.5 (1-4) | 0.216 |  | 3 (2-4) | 3 (1-3) | 0.159 |  | 3 (2 - 3) | 3.5 (1 - 4) | 0.71 |
| Pregnancy rate/cycle  (n, %) | 13/35 (37.1%) | 16/46  (34.8%) | 8/31  (25.8%) | 0.588 |  | 13/35  (37.1%) | 24/77 (31.2%) | 0.533 |  | 29/81  (35.8%) | 8/31  (25.8%) | 0.314 |

*Qualitative variables were presented by absolute and relative frequency and quantitative variables by median and 95% confidence interval. FSH: Follicle Stimulating Hormone; LH: Luteinizing Hormone; AFC: Antral Follicle Count; MII: Metaphase II oocytes.

**Supplementary Table 3.** Characteristics of women with endometriosis according to the stage of the disease and regarding the genetic models of the *FSHR:*c.2039G>A variant.

| **Variables*** | **Genetic Model of the *FSHR:*c.2039G>A (rs6166:C>T, p.Ser680Asn)** | | | | | | | | | | | |
| --- | --- | --- | --- | --- | --- | --- | --- | --- | --- | --- | --- | --- |
|  | **Additive** | | | |  | **Dominant** | | |  | **Recessive** | | |
|  | **GG** | **GA** | **AA** | **p** |  | **GG** | **GA+AA** | **p** |  | **GG+GA** | **AA** | **p** |
| **Minimal/Mild Endometriosis** | | | | | | | | | | | | |
| N | 27 (32.9%) | 41 (50.0%) | 14 (17.1%) | -- |  | 27 (32.9%) | 55 (67.1%) |  |  | 68 (82.9%) | 14 (17.1%) |  |
| LH (mUI/mL) | 4.6 (2.7-5.5) | 5.4 (4.3-6.7) | 5.1 (3.9-7.9) | 0.129 |  | 4.6 (2.7-5.5) | 5.3 (4.6-6.7) | **0.045** |  | 5 (4.3 - 5.7) | 5.1 (3.9 - 7.9) | 0.393 |
| FSH (UI/L) | 6.6 (5.4-6.9) | 7.6 (6.3-8.4) | 6.6 (4.2-9.4) | 0.152 |  | 6.6 (5.4-6.9) | 7.4 (6.3-8) | 0.140 |  | 6.9 (6.3 - 7.4) | 6.6 (4.2 - 9.4) | 0.475 |
| AFC | 9 (8-10) | 8 (6-10) | 6 (4-11) | 0.186 |  | 9 (8-10) | 8 (6-9) | 0.218 |  | 8 (8 - 10) | 6 (4 - 11) | 0.104 |
| Day of COS | 10 (9-12) | 10 (9-11) | 10.5 (9-13) | 0.299 |  | 10 (9-12) | 10 (10-11) | 0.621 |  | 10 (9 - 11) | 10.5 (9 - 13) | 0.122 |
| Total dose of rFSH (UI) | 1200  (1000-1800) | 1400  (1100-1800) | 1900  (1300-2400) | 0.104 |  | 1200  (1000-1800) | 1700  (1300-1800) | 0.237 |  | 1300  (1100 - 1800) | 1900  (1300 - 2400) | **0.040** |
| Follicles | 8 (5-12) | 7 (6-9) | 6 (3-9) | 0.274 |  | 8 (5-12) | 7 (6-9) | 0.373 |  | 7.5 (6 - 9) | 6 (3 - 9) | 0.122 |
| Oocytes | 6 (4-12) | 7 (5-9) | 5.5 (2-8) | 0.321 |  | 6 (4-12) | 7 (5-8) | 0.660 |  | 7 (6 - 8) | 5.5 (2 - 8) | 0.133 |
| MII | 6 (3-9) | 5.5 (5-7) | 4 (2-8) | 0.443 |  | 6 (3-9) | 5 (4-7) | 0.430 |  | 6 (5 - 7) | 4 (2 - 8) | 0.233 |
| Embryos | 3 (2-6) | 3 (2-5) | 3 (1-4) | 0.728 |  | 3 (2-6) | 3 (2-4) | 0.652 |  | 3 (2 - 4) | 3 (1 - 4) | 0.449 |
| Pregnancy rate/cycle  (n, %) | 8/21  (38.0%) | 14/33  (42.4%) | 6/12  (50.0%) | 0.801 |  | 8/21 (38.1%) | 20/45 (44.4%) | 0.627 |  | 22/54  (40.7%) | 6/12  (50.0%) | 0.792 |
| **Moderate/Severe Endometriosis** | | | | | | | | | | | | |
| N | 46 (35.1%) | 57 (43.5%) | 28 (21.4%) |  |  | 46 (35.1%) | 85 (64.9%) |  |  | 103 (78.6%) | 28 (21.4%) |  |
| LH (mUI/mL) | 4.8 (3.8-6.5) | 4.3 (3.8-5) | 5.2 (3.6-6.5) | 0.714 |  | 4.8 (3.8-6.5) | 4.5 (4-5.6) | 0.548 |  | 4.5 (3.9 - 5.2) | 5.2 (3.6 - 6.5) | 0.785 |
| FSH (UI/L) | 6.2 (4.5-7.6) | 7.3 (6.6-7.8) | 7.5 (6.2-8.5) | 0.199 |  | 6.2 (4.5-7.6) | 7.3 (6.7-7.8) | 0.078 |  | 6.8 (6.2 - 7.6) | 7.5 (6.2 - 8.5) | 0.323 |
| AFC | 6 (4-9) | 6 (5-8) | 6.5 (5-11) | 0.578 |  | 6 (4-9) | 6 (5-8) | 0.655 |  | 6 (5 - 8) | 6.5 (5 - 11) | 0.488 |
| Day of COS | 10.5 (10-11) | 11 (10-12) | 10 (10-11) | 0.714 |  | 10.5 (10-11) | 10.5 (10-11) | 0.715 |  | 11 (10 - 11) | 10 (10 - 11) | 0.415 |
| Total dose of rFSH (UI) | 2000  (1800-2000) | 1800  (1600-2200) | 2000  (1600-2200) | 0.924 |  | 2000  (1800-2000) | 2000  (1800-2000) | 0.859 |  | 2000  (1800 - 2000) | 2000  (1600 - 2200) | 0.694 |
| Follicles | 5 (3-6) | 6 (5-8) | 7 (5-10) | **0.029^a^** |  | 5 (3-6) | 6 (5-8) | **0.010** |  | 5 (5 - 6) | 7 (5 - 10) | 0.122 |
| Oocytes | 4 (2-6) | 5 (4-6) | 6 (5-9) | **0.029^b^** |  | 4 (2-6) | 5 (4-6) | **0.028** |  | 5 (4 - 5) | 6 (5 - 9) | **0.026** |
| MII | 4 (2-5) | 5 (4-6) | 5 (4-6) | 0.143 |  | 4 (2-5) | 5 (4-6) | 0.054 |  | 4 (4 - 5) | 5 (4 - 6) | 0.248 |
| Embryos | 2 (1-4) | 3 (2-4) | 3 (2-4) | 0.489 |  | 2 (1-4) | 3 (2-4) | 0.248 |  | 3 (2 - 3) | 3 (2 - 4) | 0.462 |
| Pregnancy rate/cycle  (n, %) | 9/40  (22.5%) | 20/47  (42.6%) | 8/25  (32.0%) | 0.139 |  | 9/40  (22.5%) | 28/72 (38.9%) | 0.077 |  | 29/87  (33.3%) | 8/25  (32.0%) | 0.901 |

*Qualitative variables were presented by absolute and relative frequency and quantitative variables by median and 95% confidence interval. FSH: Follicle Stimulating Hormone; LH: Luteinizing Hormone; AFC: Antral Follicle Count; MII: Metaphase II oocytes. ^a^No statistically significant difference was found according to genotypes comparision using Dunn Test (p>0.05). ^b^AA genotype had significantly higher oocytes retrieved compared to GG genotype using Dunn Testy (p=0.024).
